# ﻿*Hoyaspectatissima* (Gentianales, Apocynaceae), a new species from Yunnan, China

**DOI:** 10.3897/phytokeys.222.99420

**Published:** 2023-03-21

**Authors:** Bine Xue, Erfeng Huang, Gang Yao, Guohua Zhao, Jiuxiang Huang

**Affiliations:** 1 College of Horticulture and Landscape Architecture, Zhongkai University of Agriculture and Engineering, Guangzhou 510225, China Zhongkai University of Agriculture and Engineering Guangzhou China; 2 Guangxi Nanning Roy Garden Co., Ltd., Nanning 530227, China Guangxi Nanning Roy Garden Co., Ltd. Nanning China; 3 College of Forestry and Landscape Architecture, South China Agricultural University, Guangzhou 510642, China South China Agricultural University Guangzhou China; 4 Fairy Lake Botanical Garden, Shenzhen & Chinese Academy of Sciences, Shenzhen 518004, Guangdong, China Shenzhen & Chinese Academy of Sciences Shenzhen China

**Keywords:** Asclepiadoideae, Asia, Marsdenieae, morphology, nomenclature, taxonomy

## Abstract

*Hoyaspectatissima*, a new species from Yunnan Province, China, is described and illustrated. *Hoyaspectatissima* is morphologically similar to *H.lyi*, but can be easily distinguished from the latter by its succulent leaves that are 2‒4.5 cm long (vs. coriaceous leaves that are up to 9 cm long), corolla that is pink to pale pink (vs. yellowish-white) and corona lobes that are sub-rhombic in top view (vs. ovoid in top view).

## ﻿Introduction

**Figure 1.**
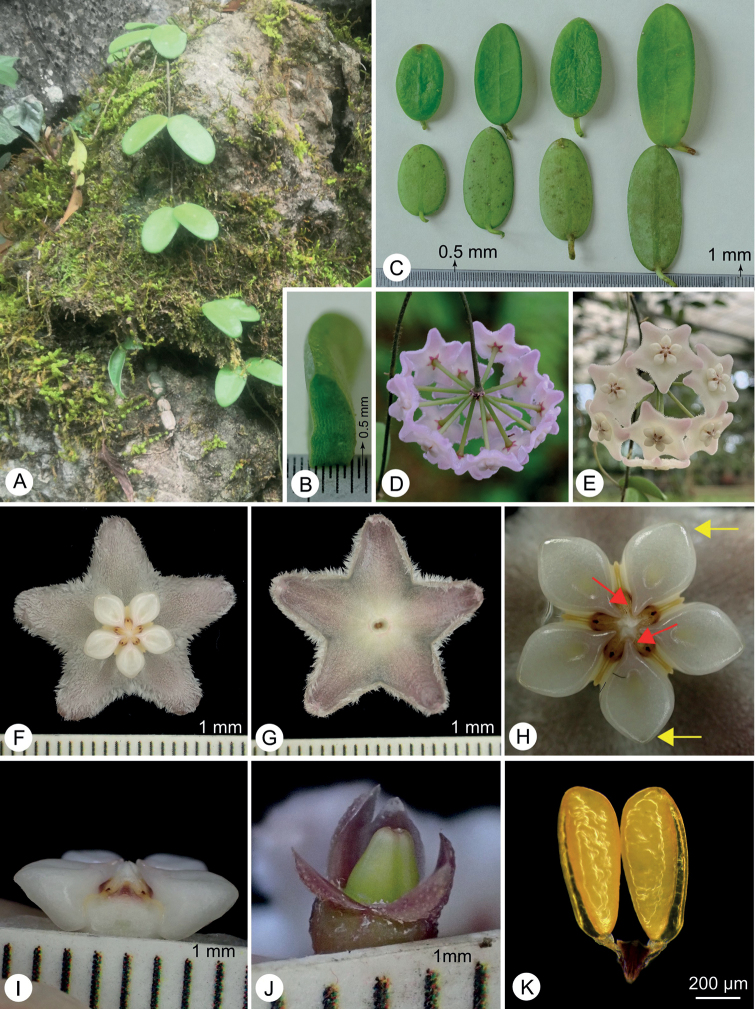
*Hoyaspectatissima***A** habit **B** cross section of lamina **C** leaves **D, E** inflorescence **F** flower (top view) **G** flower (bottom view) **H** corona (top view; Red arrowheads indicate the inner angle of the corona lobes and yellow arrowheads indicate the outer angle of the corona lobes) **I** corona (side view) **J** ovaries **K** pollinarium.

*Hoya* R. Br. is a large genus within the tribe Marsdenieae of Apocynaceae (Wanntorp et al. 2014), comprising 350‒450 species of subshrubs or lianas widely distributed in the tropical and subtropical regions of Asia, Oceania and the Pacific Islands (Li et al. 1995; Endress et al. 2018). China possesses more than 40 species (Huang et al. 2021; Huang et al. 2022), with multiple new species or newly-recorded species reported recently, such as *H.yingjiangensis* J.Feng Zhang, L.Bai, N.H.Xia & Z.Q.Peng (Zhang et al. 2015), *H.acuminata* (Wight) Benth. ex Hook.f. (Gui et al. 2017), *H.vangviengiensis* Rodda & Simonsson (Zhang et al. 2017), *H.tamdaoensis* Rodda & T.B.Tran (Nong et al. 2018), *H.burmanica* Rolfe (Ma et al. 2019), *H.longicalyx* Wang Hui & E.F.Huang (Huang et al. 2020), *H.gaoligongensis* M.X.Zhao & Y.H.Tan (Zhao et al. 2020), *H.nyingchiensis* Y.W.Zuo & H.P.Deng (Zuo et al. 2020), *H.pyrifolia* E.F. Huang (Huang et al. 2021), *H.longlingensis* E.F. Huang and *H.sichuanensis* E.F.Huang (Huang et al. 2022). Recently, we found an unknown *Hoya* species (Fig. 1) in southwest Yunnan Province, China. After a detailed morphological comparison with all the *Hoya* species recorded in China and adjacent regions, we concluded that this species is new to science. Thus, we formally describe it here.

## ﻿Taxonomic treatment

### ﻿ Hoya spectatissima

Taxon classificationPlantaeGentianalesApocynaceae

B.Xue, E.F.Huang, Gang Yao & J.X.Huang sp. nov.

1E12F3CA-A56A-5AD1-8475-E8DDE9E3156F

urn:lsid:ipni.org:names:77316067-1

Fig. 1

#### Diagnosis.

*Hoyaspectatissima* is most similar to *H.lyi* H.Lév, but differs from the latter by its succulent leaves (vs. coriaceous leaves) that are 2‒4.5 cm in length (vs. up to 9 cm in length) and rounded or rarely obtuse at the apex (vs. mostly acute at the apex), pink to pale pink corolla (vs. yellowish-white corolla) that is densely sericeous adaxially (vs. densely pubescent adaxially), ivory white corona lobes (vs. purple towards the inner angle and ivory white towards the outer angle of corona lobes) that are sub-rhombic in top view (vs. ovoid in top view) and have an acute outer angle (vs. truncate to shallowly concave outer angle).

#### Type.

China, Yunnan Province, Gengma Hsien, Sipaishan Town, Guanlong Village, Luoshuidong, on rocks in karst habitat, 7 July 2021, *E.F. Huang-RG0017* (Holotype: IBSC!).

#### Description.

Lithophytic climber. Stems rounded in cross section, up to 1 m in length, 1.5‒2 mm in diameter, densely pubescent when young, subglabrous when mature, sometimes 3‒5 branches at nodes, internodes 3‒8 cm long. Leaves opposite, wine-red and pubescent when young; mature lamina subglabrous, succulent, elliptic or oblong, 2‒4.5 × 1.3‒1.8 cm, 2.5‒3 mm in thickness, bright green adaxially, pale green abaxially, base rounded, apex rounded or rarely obtuse; mid-vein slightly elevated or obscure adaxially, not elevated abaxially, lateral veins 2‒3 pairs or sometimes invisible in adaxial surface, invisible in abaxial surface; petioles sometimes recurved, 3‒7 mm long, 1‒1.5 mm in diameter. Inflorescences axillary pseudo-umbels, many-flowered, globose; peduncle longer than pedicels, 4.5‒5 cm long, papillose to sparsely pubescent; pedicels 2.5‒3 cm long, yellow-green, papillose to sparsely pubescent. Calyx lobes triangular to narrowly triangular, ca. 1.5 × 2 mm, purplish-red, sparely pubescent or glabrous. Corolla rotate, pink in early flowering phase, pale pink in late flowering phase, ca. 2 cm in diameter, densely sericeous adaxially, sub-glabrous abaxially, lobes triangular, 5‒6 mm long and 5‒6 mm wide, margin recurved, apex rounded, revolute. Corona ivory white, 7‒8 mm in diameter, 2.0‒2.4 mm high, lobes 5, stellate spreading, ca. 3.5 × 2.5 mm, sub-rhombic in top view, outer angle acute, inner angle acuminate, the inner tips convex and spreading obviously towards the centre of the flower, ivory white or rarely shallowly pink. Pollinia clavate, ca. 0.73 × 0.26 mm, base cuneate, apex truncate, narrowing towards the base, sterile edge all along the outer edge of the pollinium, translator arms attached at the centre of the corpusculum. Ovaries 2, triangular-ovate, attached to each other, ca. 2 mm long and 1.5 mm wide, green, glabrous. Fruit and seed not seen.

#### Distribution and habit.

*Hoyaspectatissima* is known from at least two localities in Cangyuan Hsien and Gengma Hsien, Yunnan Province, China. It is a lithophytic liana that climbs on rocks in karst habitat. It is a common species in Guanlong Village, Gengma Hsien, but an occasional species in Menglai Town, Cangyuan Hsien.

#### Etymology.

*Hoyaspectatissima* is named to reflect its beautiful flowers.

#### Taxonomic discussion.

**Figure 2.**
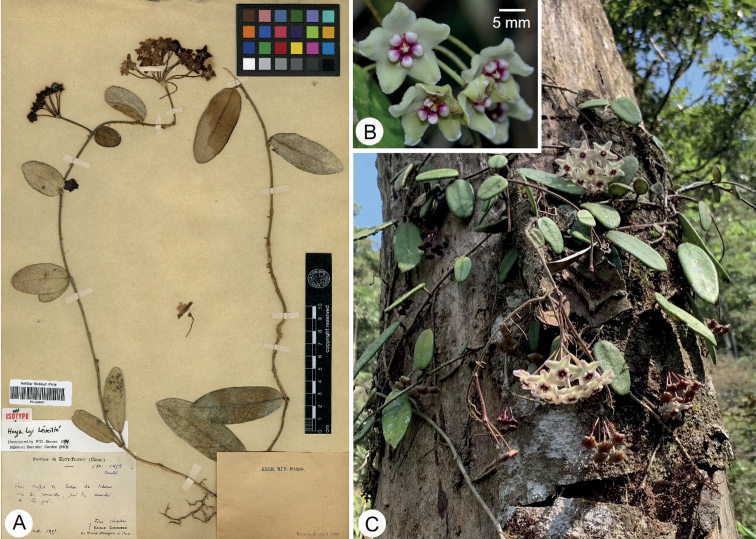
*Hoyalyi***A** isosynytpe (*E. Bodinier 1853*, P00634485) **B** inflorescence **C** habit.

*Hoyaspectatissima* has evidently succulent leaves that are 2.5‒3 mm thick (Fig. 1B); this trait is also found in *H.pandurata* Tsiang, which is also endemic in Yunnan Province, China. However, *H.pandurata* is a subshrub and is epiphytic on trees in open or mixed woods and it further differs from *H.spectatissima* by its pandurate or oblong leaves (vs. elliptic or oblong) with acuminate apex (vs. rounded or rarely obtuse), shorter peduncle and pedicels that are ca. 3 mm long (vs. 4.5‒5 cm long) and 1.5 cm long (vs. 2.5‒3 cm long) respectively and yellow or reddish corolla (vs. pink or pale pink) that is 0.8‒1 cm (vs. ca. 2 cm) in diameter (Li et al. 1995). Additionally, *H.spectatissima* is morphologically similar to *H.lyi*, a species recorded from China, Laos and Vietnam (Rodda 2012). Both species have elliptic or oblong leaves (Figs 1A, C, 2A, C), triangular to narrowly triangular calyx lobes, corolla ca. 2 cm in diameter and clavate pollinia. However, *Hoyaspectatissima* differs from *H.lyi* by the following characters: succulent leaves that are 2.5‒3 mm thick (Fig. 1B) (vs. coriaceous leaves that are less than 1.5 mm thick, Fig. 2A, C); shorter leaves that are 2‒4.5 cm long (Fig. 1C) (vs. up to 9 cm long, Fig. 2A); apex of leaves rounded or rarely obtuse (Fig. 1A, C) (vs. mostly acute, Fig. 2A, C); 2‒3 paired lateral veins in leaves (vs. 3‒5 paired lateral veins); pink to pale pink corolla (Fig. 1D, E) (vs. yellowish-white, Fig. 2B); corolla lobes densely sericeous adaxially (Fig. 1F, G) (vs. densely pubescent adaxially, Fig. 2B); corona lobes sub-rhombic in top view (Fig. 1F, H) (vs. ovoid, Fig. 2B) and ivory white (Fig. 1F, H) (vs. purple towards the inner angle and ivory white towards the outer angle, Fig. 2B); and outer angle of corona lobes acute (Fig. 1H) (vs. truncate to shallowly concave, Fig. 2B).

#### Paratype.

China. Yunnan Province, Cangyuan Hsien, Menglai Town, on rocks in karst habitat, 22 August 2021, *E.F. Huang-RG0033* (IBSC).

## Supplementary Material

XML Treatment for Hoya spectatissima
